# Revealing New Trends in the Global Burden of Hepatocellular Cancer Related to Hepatitis C Virus by Region, Sociodemographic Index, and Sex

**DOI:** 10.3390/jcm14176006

**Published:** 2025-08-25

**Authors:** Lynette Sequeira, Xiaohan Ying, Nazli Begum Ozturk, Deirdre Reidy, Arun B. Jesudian, Ahmet Gurakar

**Affiliations:** 1 Department of Internal Medicine, School of Medicine, Johns Hopkins University, Baltimore, MD 21218, USA; lsequer1@jh.edu; 2 Division of Gastroenterology and Hepatology, Weill Cornell Medicine, New York, NY 10065, USA; xiy4001@nyp.org; 3 Division of Gastroenterology and Hepatology, Saint Louis University Medical School, St. Louis, MO 63104, USA; nazlibegumozturk@gmail.com; 4 Department of Internal Medicine, Weill Cornell Medicine, New York, NY 10065, USA; dif9035@nyp.org; 5 Division of Gastroenterology and Hepatology, School of Medicine, Johns Hopkins University, Baltimore, MD 21218, USA; aguraka1@jh.edu

**Keywords:** hepatocellular cancer, hepatitis C virus, global burden of disease

## Abstract

**Background/Objectives**: Hepatocellular carcinoma (HCC) remains a leading cause of global cancer mortality, with increasing incidence and persistently poor survival. Hepatitis C virus (HCV) is a major risk factor for HCC, and while the advent of direct-acting antivirals (DAAs) has significantly altered HCV-related hepatocellular cancer (HCC-HCV) risk, the global burden remains substantial. With the World Health Organization (WHO) aiming to reduce hepatitis-related deaths by 2030, we set out to evaluate global HCC-HCV trends from 1990 to 2021, stratified by sex, WHO region, and sociodemographic index (SDI), using data from the Global Burden of Disease (GBD) 2021 study. **Methods**: We analyzed age-standardized incidence (ASI), deaths, and disability-adjusted life years (DALYs) due to HCV-HCC from 1990 to 2021 using GBD 2021 data. Trends were stratified by WHO region, sociodemographic index (SDI), and sex. Joinpoint regression modeling was used to identify statistically significant temporal inflection points and calculate the annual percent change (APC) in unique time segments and average annual percent change (AAPC) over the entire study period (1990 to 2021). **Results**: Globally, deaths and DALYs attributable to HCV-HCC increased over the study period while ASI declined modestly. The region of the Americas exhibited the highest AAPC in all three metrics, potentially driven by an aging HCV-infected population, rising comorbidities (e.g., obesity, diabetes), and improved case detection. Nevertheless, on a global level, high-SDI regions showed the most favorable trends, particularly after 2016, likely reflecting the earlier adoption of DAAs and a differential success of WHO goals. Lower-SDI regions continued to exhibit increasing burden. Notably, ASI began to rise again between 2019 and 2021, suggesting an ongoing need to critically evaluate and restructure our approach to reducing HCV and HCV-HCC. **Conclusions**: Our findings underscore the urgent need for equity-driven, region-specific strategies to achieve better control of this highly morbid disease.

## 1. Introduction

Hepatocellular cancer (HCC) continues to contribute a significant mortality burden worldwide with a continually rising worldwide incidence over the past several decades [1]. It is one of the deadliest solid tumors, with an overall 5-year survival of less than 22 percent, making the rising incidence of the disease even more concerning.

Hepatitis C virus (HCV) is an established, prominent risk factor for the development of chronic liver disease and consequently HCC [2]. The global burden of chronic hepatitis C increased by 20.4% from 1990 to 2021, with incident cases rising from 3.7 million to 4.5 million annually [3]. However, the age-standardized incidence rate (ASI) of chronic hepatitis C declined by 0.55% per year, reflecting improvements in prevention and treatment in some regions, especially high-income countries with robust viral hepatitis elimination programs. Despite these improvements, the burden of HCV remains disproportionately high in low-sociodemographic-index (SDI) countries and among older adults, with new cases in those over 70 years increasing significantly due to population aging.

According to the American Association for the Study of Liver Diseases (AASLD), 80 percent of HCC cases occur in patients with underlying cirrhosis [4]. Historically, HCV has been implicated as the most common cause of cirrhosis and consequently HCC, at least in the United States. However, the advent of direct-acting antivirals (DAAs) significantly altered the cirrhosis and HCC disease landscape [5]. It has previously been noted that achieving sustained virologic response (SVR) with DAAs can result in up to a 70 percent risk reduction for HCC in HCV patients, though this risk reduction depends on the severity of disease caused by HCV. Specifically, patients with preexisting HCV cirrhosis do remain at increased risk of HCC for up to 10 years after SVR [6,7]. Even within the population of patients with HCV cirrhosis, the HCC risk is dependent on the severity of the disease. For example, a meta-analysis by Yew et al. in 2024 found that DAA therapy reduced HCC occurrence in non-cirrhotic patients (relative risk [RR] 0.80, 95% CI 0.69–0.92) and in cirrhotic patients (RR 0.39, 95% CI 0.24–0.64), but not in those with decompensated cirrhosis [8]. In contrast, for HCV patients without cirrhosis who achieve SVR, HCC risk is so low that surveillance has been demonstrated to cost-ineffective and is not recommended by the AASLD [7,9].

Despite these promising developments, the burden of HCV-HCC morbidity and mortality has remained significant over the past several decades [10]. Moreover, the degree of burden may vary geographically, socioeconomically, and demographically and disparities in HCC-HCV remain unexplored.

In 2016, the World Health Organization (WHO) released a framework to globally reduce hepatitis incidence and mortality by 2030 [11]. As the deadline approaches, the critical evaluation of these proposed strategies is imperative to ensure their ongoing effectiveness in a constantly evolving disease landscape. In this study, we aimed to better characterize the trends in HCC-HCV stratified by sex, WHO region, and sociodemographic index (SDI) to identify vulnerable populations in order to address disparities in HCC-HCV diagnosis and treatment.

## 2. Materials and Methods

This study utilized anonymized, publicly available information and was therefore exempt from Institutional Review Board (IRB) approval.

We utilized data from the Global Burden of Disease (GBD) 2021 study (https://vizhub.healthdata.org/gbd-results/; accessed 4 April 2025). The GBD 2021 study provides information regarding the burden of 371 diseases and injuries across 21 GBD regions and 204 countries and territories from 1990 to 2021. Specifically pertaining to the present study, the GBD provides information on the burden of HCC from a variety of etiologies, including the viral hepatitis infections, alcohol, and metabolic-associated steatotic liver disease, amongst others [11]. We focused on HCC related to HCV.

### 2.1. Study Population and Data Collection

We extracted data regarding both the number of deaths and disability-adjusted life years (DALYs) as well as the ASI attributable to HCC-HCV stratified by gender from 1990 to 2021. We recorded this information for five WHO regions (African, Eastern Mediterranean, European, American, Southeast Asia, Western Pacific) as well as globally. We also recorded this information for five SDIs (high, high–middle, middle, low, low–middle, and low). Based on the absolute metrics of deaths, DALYs, and ASI in each year, we evaluated the temporal trends in the epidemiology of HCC-HCV on a global scale over the 1990-to-2021 time period.

### 2.2. Data Analysis

To analyze temporal trends, we graphed the absolute metrics of deaths, DALYs, and ASI each year in the 1990-to-2021 time frame to provide a visual representation of the changes in these metrics over the studied time frame. In addition to this, we utilized Joinpoint regression modeling (version 4.9.1.0; National Cancer Institute, Rockville, MD, USA) [12]. This statistical method identifies points at which significant changes in trends occur (“Joinpoints”) and fits linear segments to the data using the least-squares method. This approach allowed for the detection of inflection points in disease trends. We calculated the annual percent change (APC) for each linear segment, such that APCs were calculated for segments of time within the larger 1990-to-2021 time period. For instance, if the APC for deaths was positive from 1990 to 1997, but negative from 1998 to 2007, separate APCs would be reported for each of these segments (e.g., 1.5 for 1990 to 1997 and −1.5 for 1998 to 2007). The length of the time segments within the larger 1990-to-2021 period was dependent on when the inflection point occurred, not a fixed interval of time. Given the high volume of data we analyzed during this study, we focused our results on reporting the APC during and following the year 2016 to evaluate the effects of the WHO guidelines and the recent approval of DAAs.

In addition to determining the APC of various time segments based on inflection points in trends of the various metrics (deaths, DALYs, and ASI), Joinpoint also provided an overall average annual percent change (AAPC) over the entire study period (1990-to-2021). Statistical significance was determined by comparing the AAPC to zero, with a *p*-value of less than 0.05 being considered significant.

## 3. Results

We evaluated the trends in HCC-HCV in the aforementioned WHO regions and SDIs, stratified by sex. A compilation of all results, stratified by WHO region and sex, is found in Table 1 and Table 2.

### 3.1. Global Trends

Globally, from 1990 to 2021, the absolute number of deaths and DALYs for HCC-HCV increased for both males and females. In contrast, global ASI rates declined over the same period, suggesting that population growth and aging contributed to the increased absolute burden, despite improvements in age-adjusted outcomes.

### 3.2. Trends by WHO Region

#### 3.2.1. DALYs

**Figure 1:**
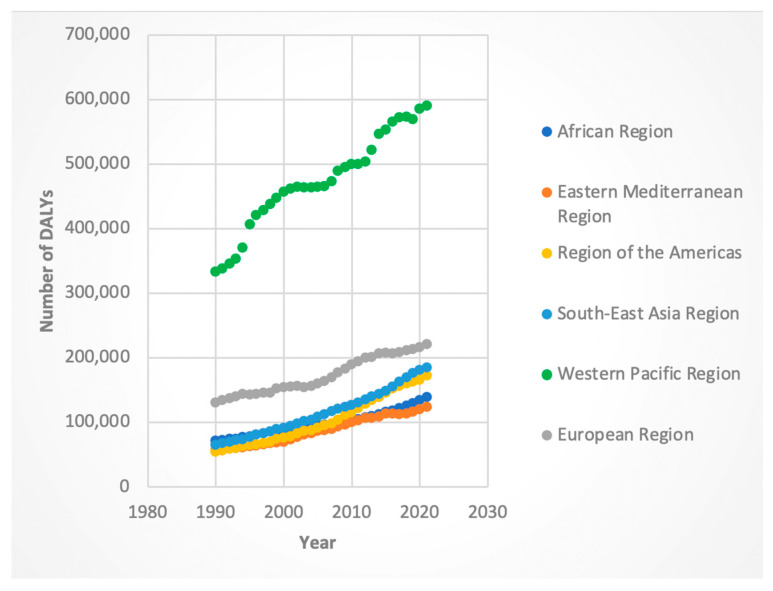
Female disability-adjusted life years (DALYs) attributable to Hepatitis-C-related hepatocellular cancer by WHO regions.

**Figure 2:**
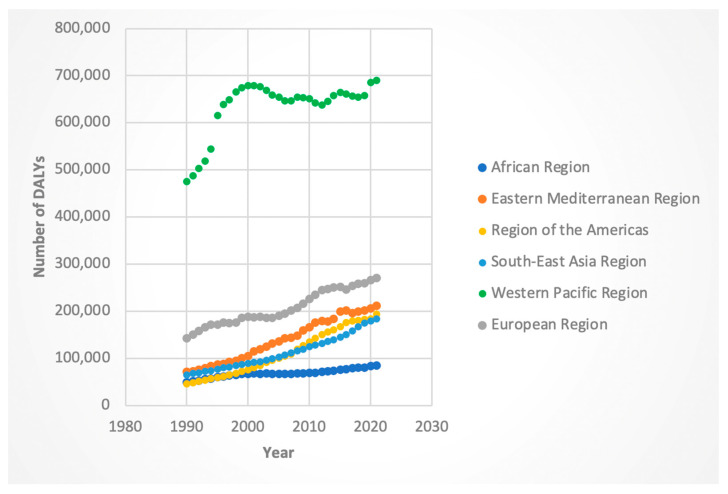
Male disability-adjusted life years (DALYs) attributable to Hepatitis-C-related hepatocellular cancer by WHO regions.

For both sexes, the AAPC in DALYs was positive across all WHO regions. Among females, the regions of the Americas (AAPC 3.73%, 95% CI: 3.63–3.81) and Southeast Asia (AAPC 3.43%, 95% CI: 3.39–3.46) exhibited the highest increases. The lowest increase was observed in the European region (AAPC 1.65%, 95% CI: 1.61–1.70). Male DALYs followed a similar pattern, with the region of the Americas showing the steepest increase (AAPC 4.63%, 95% CI: 4.55–4.70), and the Western Pacific region the lowest (AAPC 1.17%, 95% CI: 1.08–1.27). While the aforementioned areas had AAPC in DALYs, the region with the highest absolute number of DALYs for both sexes was the Western Pacific region (see Figure 1 and Figure 2).

We evaluated specific “Joinpoints” or statistically significant inflection points in trends, with a focus on the time segment containing and following 2016. For females, the region with the greatest APC in DALYs was the Southeast Asia region (APC 4.21 from 2014 to 2019) while the region with the lowest APC in DALYs was the Eastern Mediterranean region (APC −0.097 for the 2015-to-2018 period). Males exhibited a similar trend, with 2016 containing APC being greatest in the Southeast Asia region (5.04 from 2015 to 2019); however, the lowest APC was in the Western Pacific region (APC 0.68 in the 2012-to-2021 time segment).

#### 3.2.2. Deaths

For female deaths, the global AAPC was 2.71% (95% CI: 2.67–2.75), with the highest regional increase in the Americas (AAPC 3.93%, 95% CI: 3.87–3.99). Males showed similar regional disparities, with the Americas (AAPC 4.81%, 95% CI: 4.74–4.87) and Eastern Mediterranean (AAPC 3.56%, 95% CI: 3.43–3.64) contributing the most. The lowest increase in male deaths was in the African region (AAPC 1.83%, 95% CI: 1.80–1.84).

**Figure 3:**
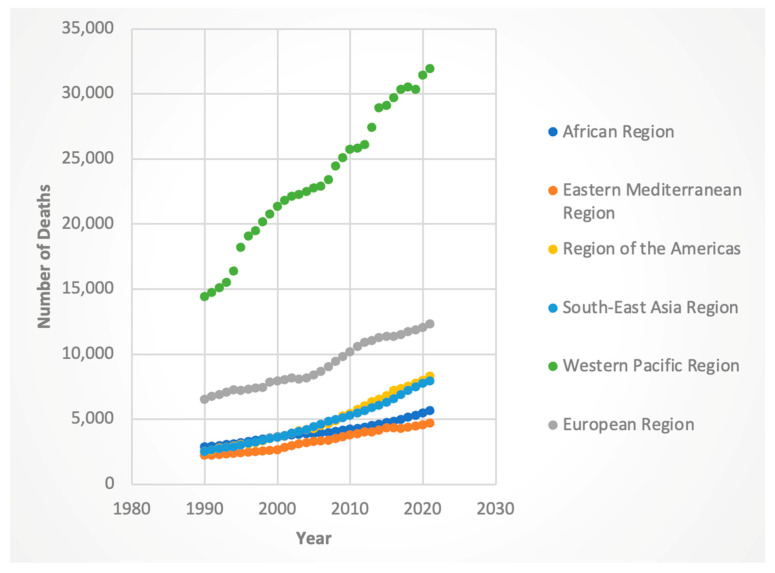
Female deaths attributable to Hepatitis-C-related hepatocellular cancer by WHO region.

**Figure 4:**
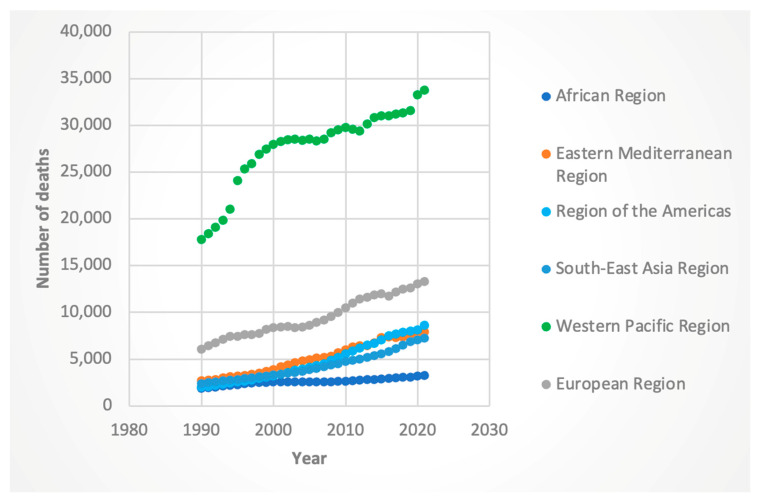
Male deaths attributable to Hepatitis-C-related hepatocellular cancer by WHO region.

For the 2016 containing Joinpoint, the WHO regions with the highest APC for deaths were the African region for females (APC was 2.45 for the 2011-to-2017 segment and 3.25 for the 2017-to-2021 segment), region of the Americas for both genders (for females, APC was 3.41 for the 2013 to 2021 segment; for males, it was 2.76 from 2016 to 2021), and Southeast Asia for both genders (for females, APC 4.44 for the 2014-to-2019 time segment; for males, 5.45 from 2015 to 2019). The lowest APC was in the Eastern Mediterranean region for females (APC was 0.40 for the 2015-to-2018 segment) and the Western Pacific region for males (APC was 0.68 from 1999 to 2019). These results can be seen in Figure 3 and Figure 4.

#### 3.2.3. ASI Rates

Among females, the global ASI rate declined (AAPC −0.51%, 95% CI: −0.53 to −0.51). ASI rates decreased in the Eastern Mediterranean, Western Pacific, and globally, but rose modestly in the European (AAPC 0.80%; 95% CI: 0.78 to 0.81), Southeast Asian (AAPC 0.32%, 95% CI: 0.30 to 0.33), and American (AAPC 1.5%, 95% CI: 1.49 to 1.51) regions. Male ASI rates showed mixed trends: increasing in the Americas (AAPC 2.20%, 95% CI: 2.15 to 2.26), Europe (AAPC 1.14%; 95% CI: 1.14 to 1.17), and Southeast Asia (AAPC 0.68%, 95% CI: 0.66 to 0.69), but declining in Africa (AAPC −0.64%, 95% CI: −0.67 to −0.62) and Western Pacific (AAPC −0.57%, 95% CI: −0.61 to −0.53).

**Figure 5:**
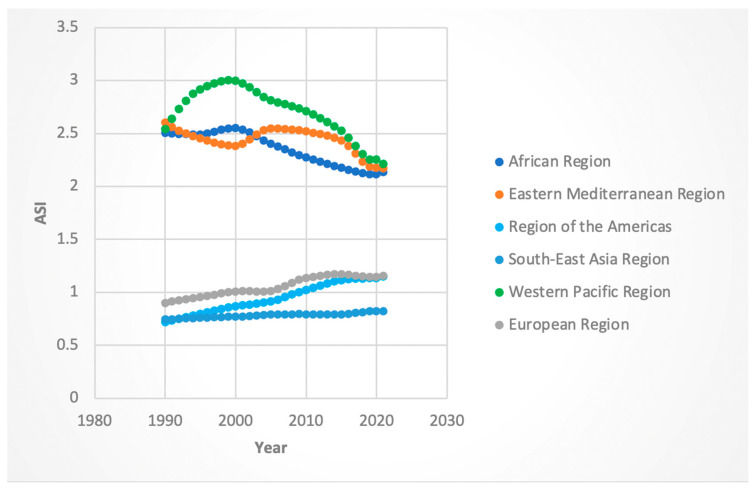
Female age-standardized incidence (ASI) of Hepatitis-C-related hepatocellular cancer by WHO region.

**Figure 6:**
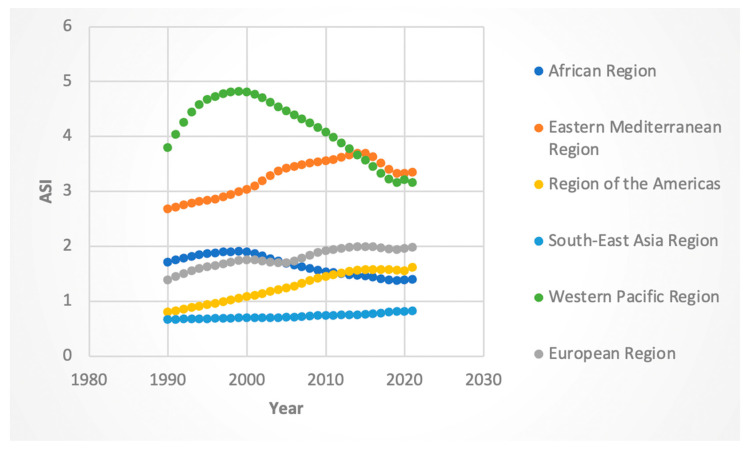
Male age-standardized incidence (ASI) of Hepatitis-C-related hepatocellular cancer by WHO region.

When considering statistically significant inflection points or Joinpoints, for females, the Western Pacific region (APC was −2.71 for the 2014-to-2019 time range) and Eastern Mediterranean (APC −2.75 for the 2015-to-2019 time range) had the greatest absolute decreases in ASI in the time period containing and following 2016. The male APC followed a similar trend, with the same two regions having the greatest absolute negative change in APC in the time period containing and following 2016 (the Western Pacific APC was −2.86 for the 2010 to 2019 time period and Eastern Mediterranean APC was −2.74 for the 2015-to-2019 time period). Though not as marked, the African region also experienced a negative APC in the Joinpoint segment containing 2016 (−0.75 from 2014 to 2019 for females and −1.29 from 2008 to 2019 for males). The results are shown in Figure 5 and Figure 6.

### 3.3. Trends by Sociodemographic Index (SDI)

#### 3.3.1. DALYs

Female DALYs increased across all SDI levels, with the highest growth in the middle–low-SDI (AAPC 2.76%, 95% CI: 2.72–2.79) and middle-SDI (AAPC 2.49%, 95% CI: 2.43–2.55) groups. Males showed the greatest increases in the same categories, particularly middle–low SDI (AAPC 3.19%, 95% CI: 3.11–3.27).

**Figure 7:**
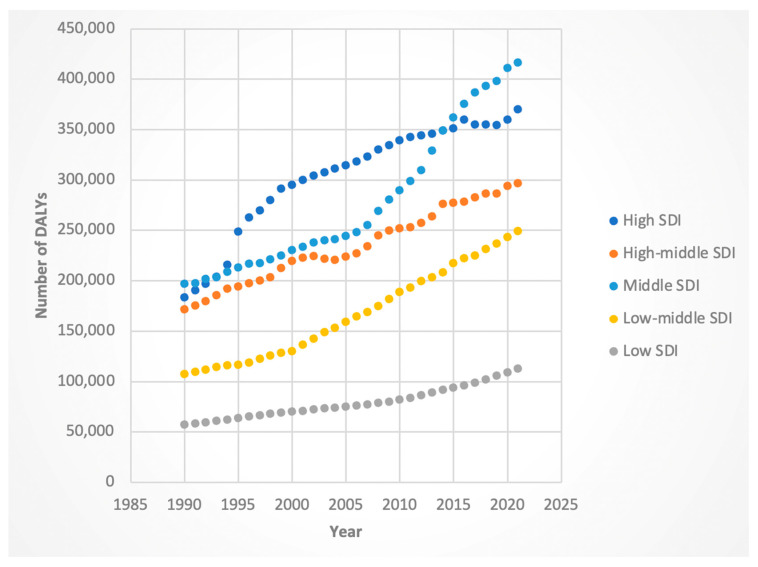
Female disability-adjusted life years (DALYs) attributable to Hepatitis-C-related hepatocellular cancer by sociodemographic index.

**Figure 8:**
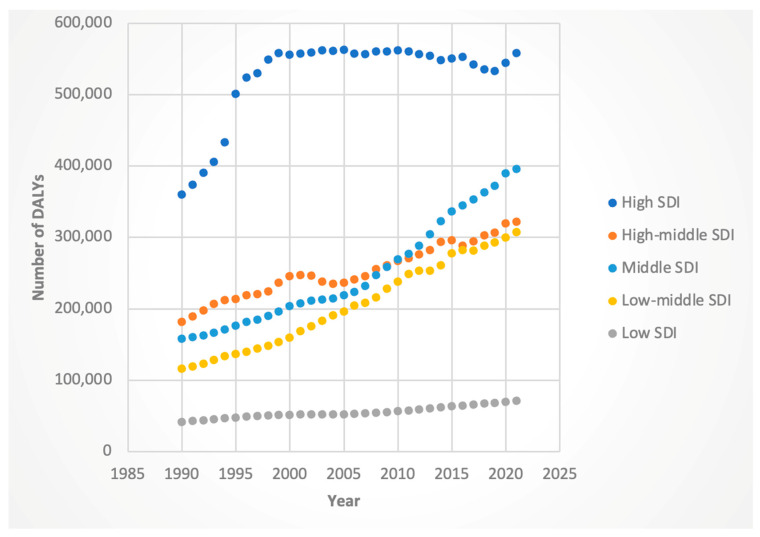
Male disability-adjusted life years (DALYs) attributable to Hepatitis-C-related hepatocellular cancer by sociodemographic index.

For the 2016 period containing Joinpoint, almost all SDIs had positive APCs. Nevertheless, high-SDI regions exhibited the most mildly positive APC for DALYs (female APC 0.61 from 2011 to 2021, male APC −0.19 from 2001 to 2021). Of note, the male APC for DALYs in the high-SDI regions was the only negative APC across all SDIs/genders for the Joinpoints containing 2016. These results are shown in Figure 7 and Figure 8.

#### 3.3.2. Deaths

**Figure 9:**
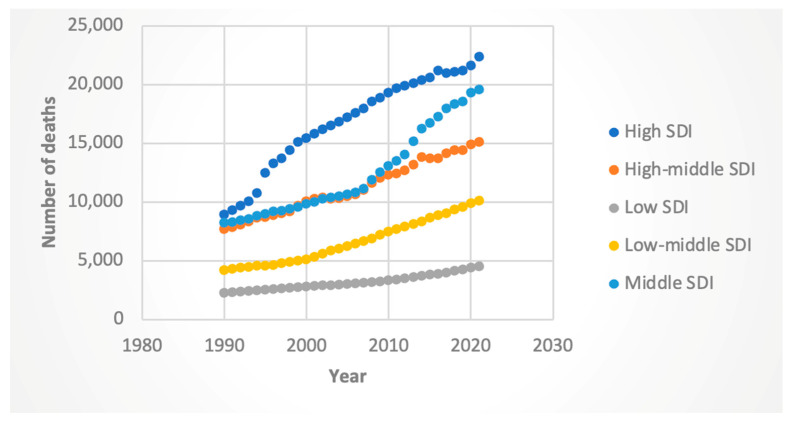
Female deaths attributable to Hepatitis-C-related hepatocellular cancer by sociodemographic index.

**Figure 10:**
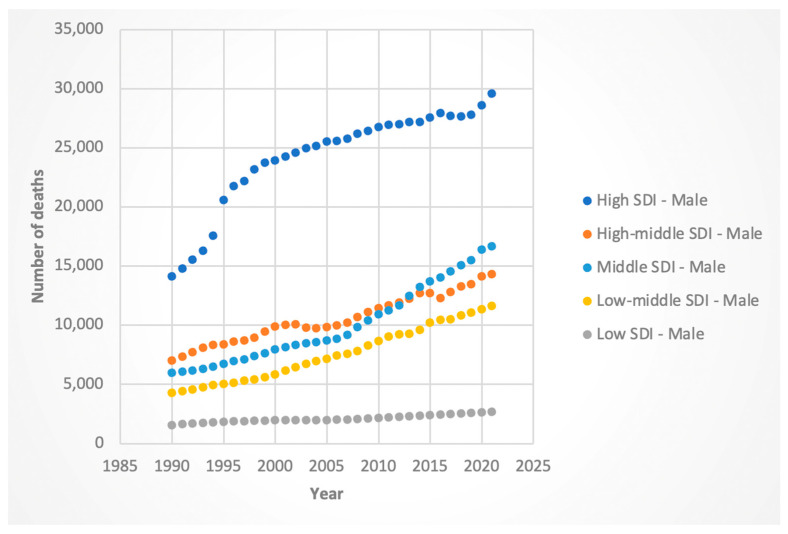
Male deaths attributable to Hepatitis-C-related hepatocellular cancer by sociodemographic index.

Female death rates rose across all SDIs, with the steepest increase in high-SDI countries (AAPC 2.94%, 95% CI: 2.86–3.02). Male deaths rose most sharply in middle-SDI (AAPC 3.39%, 95% CI: 3.33–3.46), followed closely by middle–low-SDI (AAPC 3.28%, 95% CI: 3.19–3.36), countries. These results are shown in Figure 9 and Figure 10.

#### 3.3.3. ASI Rates

Female ASI rates increased in high- (AAPC 0.97%; 95% CI 0.94 to 1.0) and high–middle (AAPC 0.11%, 95% CI 0.09 to 0.13)-SDI regions but declined in middle (AAPC −0.35%, 95% CI −0.38 to −0.33)-, middle–low (AAPC −0.23%; 95% CI −0.24 to −0.22)-, and low-SDI (AAPC −0.47%; 95% CI −0.48 to −0.46) regions. Male ASI rates increased modestly in all but the low-SDI group, which exhibited a substantial decline (−0.73%, 95% CI: −0.75 to −0.71).

**Figure 11:**
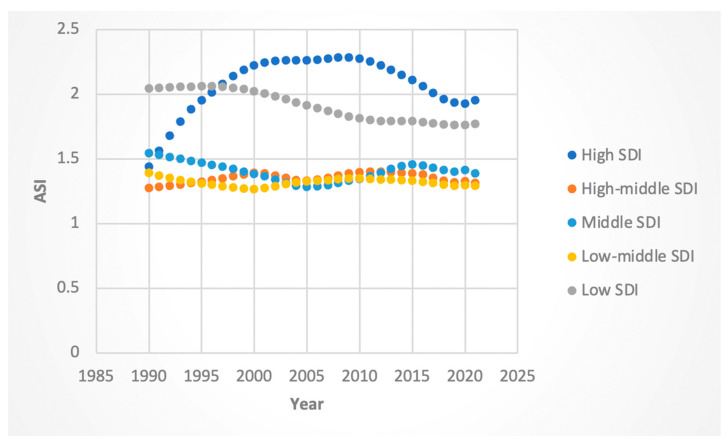
Female age-standardized incidence (ASI) of Hepatitis-C-related liver cancer by sociodemographic index.

**Figure 12:**
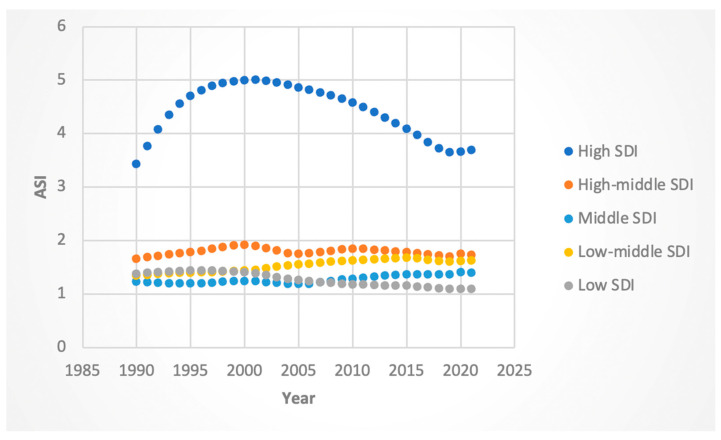
Male ASI attributable to Hepatitis-C-related hepatocellular cancer by sociodemographic index.

When considering certain “Joinpoints,” or statistically significant inflection points, high-SDI regions experienced a negative APC in ASI in the time range encompassing 2016 for both genders (−2.20 from 2014 to 2019 for females, −2.8 from 2013 to 2019 for males). The absolute value of the negative decrease in the APC of ASI in the time range encompassing and proceeding 2016 was highest for high-SDI regions for both males and females. These results are shown in Figure 11 and Figure 12.

## 4. Discussion

The global burden of HCC-HCV has continued to rise in terms of related deaths and DALYs between 1990 and 2021 despite a concurrent decline in age-standardized incidence (ASI) rates. This discordance likely reflects the combined effects of global population growth and aging, offset by improved prevention strategies, increased awareness, and the implementation of effective treatments such as DAAs [13]. As HCC is a known complication of chronic HCV infection, it is possible that the coming years will reveal decreased deaths and DALYs from HCC-HCV given this decreasing trend in incidence. Furthermore, these findings may suggest a partial success in achieving the aims of the WHO’s Global Health Sector Strategy on Viral Hepatitis.

When stratified by SDI and WHO region, nuanced patterns in HCC-HCV emerge. High-SDI countries experienced a notably steep decline in ASI in the time period containing and following 2016 (APC for ASI was −2.20 in the 2014–2019 time range for females and APC for ASI −2.80 in the 2013–2019 time range for males). The absolute value of this negative APC in the 2016-containing Joinpoint was greatest for the high-SDI regions as compared to any other SDI regions. This suggests that higher resource availability and more well-established infrastructure are important prerequisites to enacting the success of guidelines such as those put forth in 2016 by the WHO. This has indeed been confirmed by prior studies, where regions with more established infrastructure and higher resource availability (such as Iceland) were few in number but were the only ones with hopes of achieving the WHO’s hepatitis elimination targets [3,14,15,16]. Various strategies have been suggested to overcome this at the population level. For instance, the Netherlands implemented expanded and funded testing and treatment for HCV in men coinfected with human immunodeficiency virus (HIV) and leveraging preexisting HIV programs in lower-SDI regions may be a useful avenue to establish HCV care as well [17,18,19]. Additionally, systematic testing protocols implemented into the primary or preventative care setting has been shown to be useful; for example, Australia implemented systematic testing and treating HCV in prisons. Finally, expanding affordable access to DAAs is imperative [20]. Egypt, despite being classified as a lower-income country and having one of the highest global burdens of HCV, has advocated for and achieved an expanded coverage of DAAs [20,21,22].

Regionally, several areas contributed disproportionately to the global decline in ASI. The Western Pacific and African regions (for both sexes) and the Eastern Mediterranean region (for females) demonstrated particularly strong downward trends. It is interesting to note that for deaths and DALYs, the Western Pacific region consistently had the highest absolute numbers of these metrics (see Figure 1, Figure 2, Figure 3 and Figure 4). Nevertheless, the Western Pacific region exhibited some of the steepest declines in ASI, deaths, and DALYs during the segment containing and following 2016. This could imply a partial success of the WHO guidelines, particularly for patients who are diagnosed with HCV at an early, pre-cirrhosis stage, in the Western Pacific region that has been historically heavily burdened by the viral hepatitis infections [23]. Nevertheless, the persistent, markedly higher absolute burden of HCC-HCV in this region may be due to gaps in the diagnosis and treatment of HCV at earlier stages. Le et al. noted that in 2019, only 21% of chronic HCV infection were diagnosed and 11% treated in Asian and Pacific countries, suggesting ongoing room to improve the diagnosis of HCV and more widespread implementation of DAAs [24].

Another interesting regional trend was that of the AAPC over the entire study period in the region of the Americas. This region saw the highest AAPC in DALYs, deaths, and ASI in the 1990-to-2021 time range for both males and females. This may be related to several factors. First, there has been a recent uptick in the burden of HCV in the region of the Americas related to the increasing burden of injection drug use related to the opioid epidemic [25,26]. This may result in an even higher incidence of HCV-HCC in the coming years as increasing cases of chronic HCV may cause more cases of HCC. Furthermore, HCC risk is known to correlate with comorbid conditions like diabetes, obesity, and alcohol use [27]. All of these are increasing in the region of the Americas; however, obesity, diabetes, and other conditions falling within the metabolic syndrome window and consequently contributing to metabolic associated steatotic liver disease (MASLD), which is perhaps disproportionately increasing in the region of the Americas as compared to other global regions [28]. The co-occurrence of alternate etiologies to liver disease likely synergistically contributes to the progression of chronic liver disease in this region and subsequently increases HCC risk. Finally, in the United States, which comprises a significant portion of this region, about 70 percent of HCV patients were infected between 1970 and 1990 while DAAs were not widely implemented until late 2014 [29,30]. Finally, in comparison to other global regions, the United States has extremely robust cancer registries, possibly resulting in more HCV-HCC cases being captured and reported in this region as compared to other global regions [31,32]. In contrast, it has been previously reported that fewer than 20 percent of HCV infected individuals in Africa have received a diagnosis [33,34]. Finally, while the United States does fall into the region of the Americas and has the infrastructure to successfully roll out interventions like DAAs, other countries in this region (such as low- and middle-SDI regions like Bolivia, Honduras, Guyana, etc.) may be less well equipped [35,36].

Upon stratification by sex, we noted that the AAPCs and APCs generally exhibited more favorable trends for females as compared to males for all metrics. This may point to the utility of established, screening programs (such as prenatal ones) that women are globally more privy to as compared to males [37]. Nevertheless, the previously established gender differential of men experiencing a higher incidence and mortality burden of HCC has been established to be multifactorial, encompassing a complex interplay of hormonal effects and immunogenic differences [38,39].

To comment on immediate next steps, we also evaluated the Joinpoints of the most recent time segment (2019 to 2021). Most concerningly, during this time segment, ASI had begun to rise again globally for both females (0.59) and males (0.77). It is interesting that this time period coincided with the COVID-19 pandemic, which was known to have effects on alcohol consumption [40,41,42]. The ongoing evaluation of this database in the coming years will be imperative to continue to characterize recent trends. Ongoing critical evaluation and adjustments to global approaches to screening and surveillance are necessary in a continuously evolving disease landscape.

There are inherent limitations to the utilization of the GBD 2021 study. Many countries, especially in lower-SDI settings, lack complete population-based cancer registries; when present, data are often delayed [33,43]. Other limitations include lags in reporting, and comparatively lower representations of conflict-affected areas and mobile populations are limitations in the GBD that must be recognized. Nevertheless, the GBD remains the most comprehensive, standardized, and globally comparable source of cancer burden estimates, allowing for consistent cross-country and temporal analyses that would otherwise be impossible in the absence of complete primary data.

## 5. Conclusions

Overall, these findings reinforce the need for tailored, equity-focused strategies to address the global burden of HCV-related HCC. Perhaps most notably, our study demonstrated the differential trends of HCC-HCV in higher- versus lower-SDI regions, with high-SDI regions consistently demonstrating more favorable trends in deaths, DALYs, and ASI as compared to the lower-SDI ones. Though the HCV and HCV-HCC disease landscape is exciting and constantly developing with new, cutting-edge treatment strategies, grass root efforts to ensure equitable access to HCV and HCV-HCC screening, prevention, and treatment are imperative.

## Figures and Tables

**Table 1:** Male and female AAPC stratified by WHO region.

WHO Region	Female DALYs AAPC (95% CI)	Male DALYs AAPC (95% CI)	Female Deaths AAPC (95% CI)	Male Deaths AAPC (95% CI)	Female ASI AAPC (95% CI)	Male ASI AAPC (95% CI)
Americas	3.73 (3.63–3.81)	4.63 (4.55–4.70)	3.93 (3.87–3.99)	4.81 (4.74–4.87)	1.5 (1.49–1.51)	2.20 (2.15–2.26)
Southeast Asia	3.43 (3.39–3.46)	3.38 (3.35–3.41)	3.76 (3.71–3.79)	3.65 (3.61–3.69)	0.32 (0.30–0.33)	0.68 (0.66–0.69)
Europe	1.65 (1.61–1.70)	2.05 (1.97–2.15)	1.97 (1.90–2.02)	2.53 (2.44–2.63)	0.80 (0.78–0.81)	1.14 (1.14–1.17)
Western Pacific	1.86 (1.79–1.92)	1.17 (1.08–1.27)	2.57 (2.48–2.66)	2.12 (2.0–2.21)	−0.51 (−0.53 to −0.51)	−0.57 (−0.61 to −0.53)
Eastern Mediterranean	2.51 (2.45–2.56)	3.51 (3.37–3.62)	2.49 (2.43–2.54)	3.56 (3.43–3.64)	−0.58 (−0.60 to −0.56)	0.71 (0.68–0.73)
Africa	2.17 (2.15–2.18)	1.80 (1.79–1.82)	2.23 (2.22–2.25)	1.83 (1.80–1.84)	−0.52 (−0.53 to −0.51)	−0.64 (−0.67 to −0.62)

**Table 2:** Male and female AAPC stratified by SDI.

SDI Level	Female DALYs AAPC (95% CI)	Male DALYs AAPC (95% CI)	Female Deaths AAPC (95% CI)	Male Deaths AAPC (95% CI)	Female ASI AAPC (95% CI)	Male ASI AAPC (95% CI)
High	1.77 (1.71–1.83)	1.34 (1.25–1.43)	2.94 (2.86–3.02)	2.33 (2.24–2.40)	0.97 (0.94 to 1.0)	0.22 (0.18–0.25)
High–Middle	2.49 (2.42–2.55)	1.80 (1.73–1.91)	2.23 (2.16–2.30)	2.26 (2.13–2.37)	0.11 (0.09 to 0.13)	0.17 (0.12–0.20)
Middle	2.49 (2.43–2.55)	3.02 (2.97–3.09)	2.88 (2.81–2.94)	3.39 (3.33–3.46)	−0.35 (−0.38 to −0.33)	0.44 (0.39–0.48)
Middle-Low	2.76 (2.72–2.79)	3.19 (3.11–3.27)	2.89 (2.85–2.92)	3.28 (3.19–3.36)	−0.23 (−0.24 to −0.22)	0.61 (0.60–0.62)
Low	2.22 (2.21–2.23)	1.76 (1.74–1.78)	2.31 (2.29–2.33)	1.78 (1.76–1.80)	−0.47 (−0.48 to −0.46)	−0.73 (−0.75 to −0.71)

## Data Availability

The original contributions presented in this study are included in the article. Further inquiries can be directed to the corresponding author.

## Abbreviations

The following abbreviations have been used in this manuscript:

HCC	Hepatocellular carcinoma
HCV	Hepatitis C virus
DAA	Direct-acting antiviral
SVR	Sustained viral response
HCV-HCC	Hepatocellular cancer related to HCV
WHO	World Health Organization
SDI	Sociodemographic index
DALY	Disability-adjusted life year
GBD	Global Burden of Disease 2021 Study
ASI	Age-standardized incidence
APC	Annual percent change
AAPC	Average annual percent change
CI	Confidence interval
